# Sinonasal Neuroendocrine Carcinoma in Adult Proteus Syndrome

**DOI:** 10.22038/IJORL.2023.73128.3472

**Published:** 2023-11

**Authors:** Giorgos Sideris, Thomas Nikolopoulos, Antigone Sourla, Penelope Korkolopoulou, Pavlos Papadakis, Alexander Delides

**Affiliations:** 1 *Second * *ΕΝΤ* * Department, School of Medicine, "Attikon" University Hospital, National & Kapodistrian University of Athens, Athens, Greece.*; 2 *First Department of Pathology, School of Medicine, National & Kapodistrian University of Athens, Athens, Greece.*

**Keywords:** PTEN hamartoma tumor Syndrome, Proteus Syndrome, Sinonasal neuroendocrine carcinoma, Typical carcinoid, Otolaryngology

## Abstract

**Introduction:**

Proteus syndrome (PS) is a rare genetic disorder usually caused by mutations in AKT1 or PTEN genes, characterized by multiple, asymmetric tissue overgrowth with high clinical variability. Sinonasal neuroendocrine carcinomas (SNEC) are exceptionally rare tumors encountered in the ethmoid sinus, nasal cavity, or maxillary sinus.

**Case Report:**

We report a 35-year-old patient with PS, who underwent successful surgical removal of a well-differentiated SNEC obstructing his nasal cavity and highlight the role of the otolaryngologist for safe airway management, minimal surgical intervention and coordination of the multidisciplinary care. Histologically, focally hyperplastic mucosal epithelium of respiratory type of the nasal chamber was noticed along with seromucinous glands and capillary congestion of the subepithelial fibrovascular tissue. The limited presence of neoplastic tissue with histomorphological and immunophenotypic features of a neuroendocrine neoplasm was focally observed. Tumor cells grow in the form of islets within a vascular stroma; these neoplastic cells are immunohistochemically positive for synaptophysin, CD56, EMA, Ki67 (low expression, cell proliferation rate: 2%), CD31, chromogranin and pancytokeratin AE1 / AE3 as well as for S-100 protein (weak intensity)

**Conclusions:**

This first description of a SNEC in a PS patient, might hint towards a common basis between the two conditions, due to the mosaic AKT1 variant and an activated AKT/PIK3CA/PTEN pathway.

## Introduction

PTEN hamartoma tumor syndrome (PHTS) encompasses several clinical syndromes with germline mutations in the PTEN tumor suppressor gene, including Cowden Syndrome (CS), while Proteus Syndrome (PS) is considered a subgroup of CS characterized by multiple, asymmetric tissue overgrowth with high clinical variability (1). Sinonasal neuroendocrine carcinomas (SNEC) are exceptionally rare tumors encountered in the ethmoid sinus, nasal cavity, or maxillary sinus. Treatment strategies for SNECs depend on the stage and grade of the tumor, as well as its histologic characteristics and include surgery, postoperative chemotherapy, and radiotherapy (2). We report a 35-year-old patient with PS, who underwent successful surgical removal of a well-differentiated SNEC obstructing his nasal cavity. This is the first case of a typical carcinoid reported in PS. 

## Case Report

**Fig. 1:**
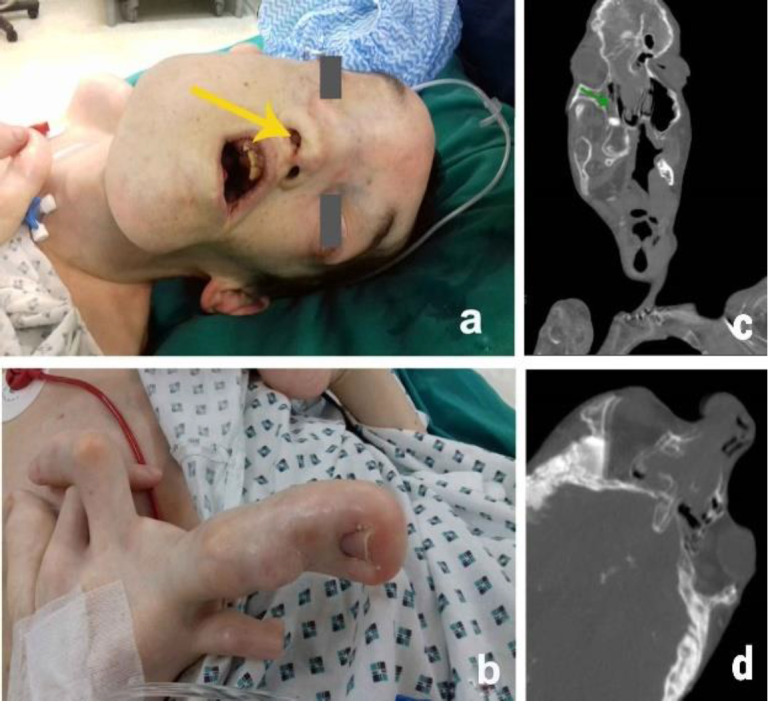
Clinical and imaging findings

A 35-year-old man with PS presented with a right nasal cavity obstruction associated with sleep apnea. A clinical examination revealed multiple abnormalities. The patient had an extensive past surgical history, including right leg amputation, inguinal hernia surgery, tonsillectomy, removal of testicular mesothelioma, mastoidectomy (brain abscess), dental extractions, and endoscopic removal of nasopharyngeal angiofibroma. A CT scan showed a protruding mass stemming from the region of the sphenopalatine foramen and localizing to the nasal cavity, maxillary sinus, and nasopharynx (Fig. 1). 

a,b: Μacrocephaly, right facial hemihypertrophy and mandibular prognathism. In the oral cavity, gingival hyperplasia was present, as well as hypoplastic haphazardly arranged teeth, reduced tongue mobility, and an open mouth at rest. Physical examination of the spine revealed severe thoracic and cervical kyphoscoliosis. Other PS characteristic features included hypertrophic auricles and enlarged hands with macrodactyly (yellow arrow: mass in the right nostril).

c,d: Computed tomography scan showing the carcinoid lesion occupying the right nasopharynx and right maxillary sinus. There is no extension into other structures such as the infratemporal fossa, buccal region, orbital cavity, despite anterior and minimal middle cranial fossa involvemen. The sinus is smaller than its contralateral counterpart and nasal passages are rotated to the left (green arrow).

The endoscopic biopsy and subsequent histologic examination revealed a malignancy with neuroendocrine features. 

A multidisciplinary medical team decided to treat the patient surgically. Preoperative fiberoptic evaluation revealed anatomic malformations of the epiglottis. Intubation was successfully performed with a fiberoptic bronchoscope. 

The tumor was resected endoscopically using the “one bleed at a time” method. Middle meatal antrostomy and ethmoidectomy were avoided due to the relatively small size of the maxillary sinus. Histologically, focally hyperplastic mucosal epithelium of the respiratory type of the nasal chamber was noticed along with seromucinous glands and capillary congestion of the subepithelial fibrovascular tissue. 

**Fig. 2:**
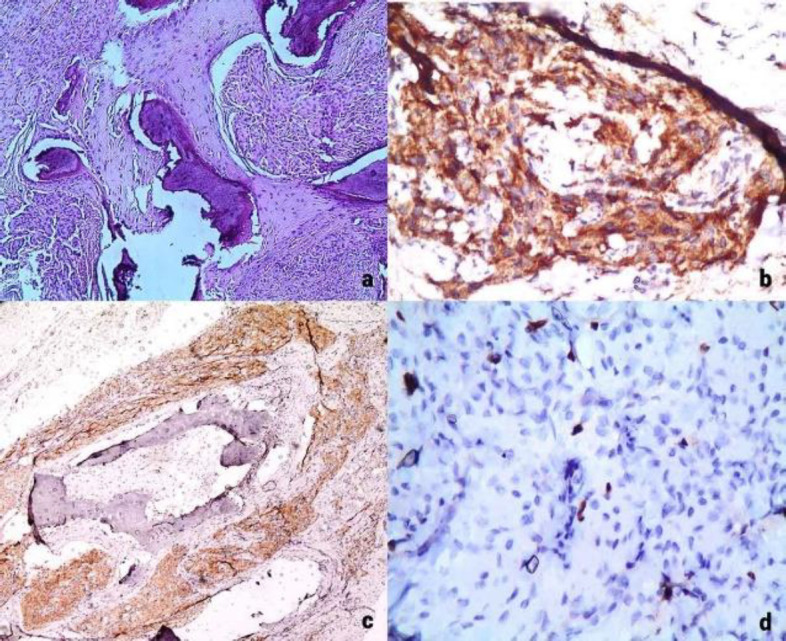
Photomicrograph of histological examination

The limited presence of neoplastic tissue with histomorphological and immunophenotypic features of a neuroendocrine neoplasm was focally observed. Tumor cells grow in the form of islets within a vascular stroma; these neoplastic cells are immunohistochemically positive for synaptophysin, CD56, EMA, Ki67 (low expression, cell proliferation rate: 2%), CD31, chromogranin and pancytokeratin AE1 / AE3 as well as for S-100 protein (weak intensity) (Fig. 2). 

a: Nests of uniform neoplastic cells around osseous trabecula (H-E x100). b: Epithelial nature of neoplastic cells is confirmed by their EMA immunoreactivity. Note the membranous accentuation of positive stain (Immunochemistry x400). C: The neuroendrocrine differentiation of neoplastic cells is confirmed, among others, with their immunostaining for synaptophysin (Immunochemistry x40). d: Low expression of Ki-67 among neoplastic cells (Immunochemistry x400).

## Discussion

First comment: Neoplasms are rare and heterogeneous in PS patients. Except for lipomas, reported neoplasms include monomorphic adenoma of the parotid gland, cystadenomas of the ovary, testicular tumors, meningiomas, and mesotheliomas (3). Patients with PHTS are at increased risk of developing cancer due to pathogenic PTEN germline variants. Hendricks et al. in their review on age-related cancer risks in PTEN hamartoma tumor syndrome report that the median age at diagnosis was 36 years while the most common tumors include female breast cancer, endometrium cancer, thyroid cancer, renal cancer, colorectal cancer and melanoma (4). 

On the other hand, Greidinger et al. report that there is a significant enrichment of Neuroendocrine Tumors (NETs) among PHTS patients, pointing to PI3K/mTOR pathway activation as important in the etiology of NET of the pancreas and other organs (5). 

While there is no good evidence that Proteus syndrome is caused by PTEN variants, there is a distinct and interesting phenotype designated as Type II Segmental Cowden syndrome that is caused by a mosaic of second-hit mutations in PTEN (with a familial PTEN variant in trans and family members with the common form of Cowden syndrome). Τhis paper demonstrates the opinion that the presence of two rare clinical entities, PS and SNEC, in the same patient may not be a coincidence, moreover, that a germline PTEN mutation may confer increased risk for NETs in addition to other tumors. 

While we cannot exclude a coincidental occurrence, we hypothesize that individuals with Proteus syndrome are susceptible to SNEC, due to the mosaic AKT1 variant and an activated AKT/PIK3CA/PTEN pathway. Further studies are needed to confirm this observation. Second comment: The most common symptoms of SNEC reported are nasal obstruction, facial pain and epistaxis. The proposed treatment should include surgery alone for resectable well-differentiated tumors (6). A meta-analysis concludes that surgery should be the cornerstone of treatment, supplemented by radiotherapy in poorly differentiated subtypes and suggests that chemotherapy does not seem to increase survival (7). 

The patient described in this report is an adult PS patient and the removal of his SNEC will improve the quality of his life, while his life expectancy is difficult to predict. He was staged as stage III according to the AJCC (T3N0M0), and was decided to be treated only surgically, via a minimal ENT intervention, since this is the main option for typical carcinoids.

Third comment: To the best of our knowledge, only ten cases of ENT surgical procedures in PS patients have been published to date including parotidectomy (two cases), thyroidectomy (one case), tonsillectomy (four cases), functional endoscopic sinus surgery (one case) and canaloplasty with tympanoplasty and mastoidectomy (two cases) (8-15). This report also aims to highlight the otolaryngologist’s crucial role at the time of airway management in individuals with PS. Due to concerns about bleeding or the inability to navigate around the obstruction, rigid bronchoscopy is preferred both for examination and intubation as in our patient. On the other hand, the need for emergent tracheostomy, cricothyroidotomy or sternal tracheotomy due to complicated larynx anatomic abnormalities and kyphoscoliosis, can arise in such cases (16).

## Conclusion

This paper demonstrates the opinion that the presence of two rare clinical entities, PS and SNEC, in the same patient may not be a coincidence, moreover, that a germline PTEN mutation may confer increased risk for NETs in addition to other tumors. Further studies are needed to confirm the hypothesis that individuals with Proteus syndrome are susceptible to SNEC due to the mosaic AKT1 variant and an activated AKT/PIK3CA/PTEN pathway.

## References

[1] Pilarski R (2009). Cowden syndrome: a critical review of the clinical literature. J Genet Couns..

[2] Chang CF, Li WY, Shu CH, Ho CY (2010). Sino-nasal neuro-endocrine carcinoma. Acta Otolaryngol..

[3] Gordon PL, Wilroy RS, Lasater OE, Cohen MM Jr (1995). Neoplasms in Proteus syndrome. Am J Med Genet..

[4] Hendricks LAJ, Hoogerbrugge N, Schuurs-Hoeijmakers JHM, Vos JR (2021). A review on age-related cancer risks in PTEN hamartoma tumor syndrome. Clin Genet..

[5] Greidinger A, Miller-Samuel S, Giri VN, Woo MS, Akumalla S, Zeigler-Johnson C, Keith SW, Silver DP (2020). Neuroendocrine Tumors Are Enriched in Cowden Syndrome. JCO Precis Oncol..

[6] Mehta GU, Raza SM, Su SY, Hanna EY, DeMonte F (2020). Management of olfactory neuroblastoma, neuroendocrine carcinoma, and sinonasal undifferentiated carcinoma involving the skullbase. J Neurooncol..

[7] van der Laan TP, Iepsma R, Witjes MJ, van der Laan BF, Plaat BE, Halmos GB (2016). Meta-analysis of 701 published cases of sinonasal neuroendocrine carcinoma: The importance of differentiation grade in determining treatment strategy. Oral Oncol..

[8] Zingade ND, Zingade NN (2008). A rare case of Proteus syndrome. J Laryngol Otol..

[9] Delides A, Panayiotides JG, Kaberos A, Giotakis I (2017). Nasopharyngeal angiofibroma in an adult with Proteus syndrome. First reported case. Hippokratia..

[10] Gordon PL, Wilroy RS, Lasater OE, Cohen MM Jr (1995). Neoplasms in Proteus syndrome. Am J Med Genet..

[11] Sahni JK, Kumar S, Wadhwa V, Kathuria G (2006). Proteus syndrome with huge tonsillar mass causing dysphagia: a rare case. J Laryngol Otol..

[12] Cantone E, Cavaliere M, Castagna G, Marino A, Del Vecchio L, Iengo M (2015). Operative Management of OSAS in a Complex Case of Proteus Syndrome. Case Rep Otolaryngol..

[13] Salinas CR, Nuyen BA, Jafari A, Nation J (2017). Refractory sleep-disordered breathing due to unilateral lingual tonsillar hypertrophy in a child with Proteus Syndrome. Int J Pediatr Otorhinolaryngol..

[14] Doherty JK, Maceri DR (2005). Ossicular discontinuity and exostoses in Proteus syndrome: a case report. Ann Otol Rhinol Laryngol..

[15] Stozhkova IV, Pchelenok EV, Kosyakov SY (2020). Sindrom Proteya v praktike otorinolaringologa: klinicheskii sluchai [Proteus syndrome in the practice of an otorhinolaryngologist: a clinical case]. Vestn Otorinolaringol..

[16] Patel KG, Zdanski CJ (2008). Cricothyroidotomy vs sternal tracheotomy for challenging airway anatomy. Laryngoscope..

